# Vitamins and Prostate Cancer Risk

**DOI:** 10.3390/molecules15031762

**Published:** 2010-03-12

**Authors:** Krishna Vanaja Donkena, R. Jeffrey Karnes, Charles Y.F. Young

**Affiliations:** Mayo Clinic College of Medicine, Rochester, MN 55905, USA

**Keywords:** vitamins A, B, C, D, and E, folic acid, retinoids, SNP, prostate cancer

## Abstract

Prostate cancer (PC) is the second most common cancer in men worldwide. Its prevention and treatment remain a challenge to clinicians. Here we review the relationship of vitamins to PC risk. Many vitamins and related chemicals, including vitamin A, retinoids, several B vitamins, vitamin C, vitamin D and vitamin E have shown their anti-cancer activities as anti-oxidants, activators of transcription factors or factors influencing epigenetic events. Although laboratory tests including the use of animal models showed these vitamins may have anti-PC properties, whether they can effectively prevent the development and/or progression of PC in humans remains to be intensively studied subjects. This review will provide up-to-date information regarding the recent outcomes of laboratory, epidemiology and/or clinical trials on the effects of vitamins on PC prevention and/or treatment.

## Introduction

According to the American Cancer Society, the estimated deaths and newly diagnosed cases of prostate cancer (PC) in the USA in 2009 will be 27,360 and 192,280 men, respectively,. Inspired by the findings that Asian men with low PC incidence and mortality rates in their birth places would have significantly higher rates after migrating to western countries, it was realized that lifestyle and dietary factors may be crucial for such changes. These above observations with additional epidemiologic and laboratory studies in life style, nutrition and diet seem to help to form concepts of cancer chemoprevention, which is a strategy seeking reduction of cancer risk by use of chemical agents [1,2,3,4,5,6]. These agents should possess abilities to prevent, delay or reverse cancer formation as well as progression. Indeed, PC can be an ideal cancer target for chemoprevention strategies, because of its long latency of disease onset, high incidence and mortality rates. It has been suggested that proper diets may eventually reduce 50–60% incidence of many types of cancer including PC. Moreover, the total medical expenditure for PC treatment has been estimated as $1.3 billion in 2000, which represents a 30% increase compared to that in 1994. In 2004, 2.3 billion was estimated for PC alone [7]. As being a prevalent cancer disease in men, today’s total cost for PC treatments in this country would exceed $2.3 billion. Therefore, the potential impact of PC chemoprevention could be enormous, with respect to PC patients, in saving life, increasing quality of life and reducing financial burden of the society.

In the recent years, because of increasing in awareness of usage of complementary and alternative medicines in health management and believing that “natural” chemicals are always safe, it has been indicated that consumption of vitamins, minerals and other dietary supplements in general population and in those who have diagnosed for cancer or other chronic diseases is increased [8,9,10,11,12]. Studies [8,9,11,13] showed that men diagnosed with PC had a higher rate of taking the supplements than that in healthy men in the general population. Perhaps, men being diagnosed with PC or at high risk for developing PC may have strong motivation to use the supplements to improve their health conditions. However, the general public comsumers and researchers may have to deal with massive information with inconsistent, conflicting or immature advice regarding the use of the supplements as means for cancer prevention strategies. This article is not to provide guidance for how to choose and use vitamin supplements but to gather up-dated information about what the vitamins currently stand in terms of their anti-PC potencies in laboratory and epidemiological as well as in some clinic trial studies.

Vitamins are a group of structurally and functionally unrelated natural chemicals that are essentially needed for normal physiology including body growth, development and metabolic functions. A well balanced, healthy food would support all of the vitamins as needed in bodies. Whether deficiencies or excessive but tolerable supplies of vitamins have effects on risk for PC development or progression will be subjected to discussion in this article.

## Vitamin A and Related Compounds

Carotenoids, retinoids and vitamin A are a group of structurally and metabolically related chemicals, many of which have been studied for their potential effects on PC chemoprevention. Therefore we will discuss their effects in PC prevention together.

Carotenoids may exist in animals and plants, but are only synthesized in plants and some microorganisms including bacteria, yeasts, and molds. Animals and humans do not have ability to synthesize carotenoids de novo, thus depending on dietary supply. Carotenoids may be divided into two main groups, *i.e.*, carotenes and xanthophylls [14,15]. β-Carotene, α-carotene, and lycopene are important members of the carotene group. Zeaxanthin, lutein, α-and β-cryptoxanthin, canthaxanthin and astaxanthin are the major xanthophylls. Moreover, about 50 different carotenoids [16] including α-carotene, β-carotene and β-cryptoxanthin, are viewed as pro-vitamin A, can be converted to vitamin A such as retinol, retinal or retinoic acid [14,15]. The conversion of a pro-vitamin A carotenoid like β-carotene to retinal occurs in the small bowel mucosa as well as in the liver by β,β-carotene-15,15′ monooxygenase (previously termed β-carotene-15,15′ dioxygenase) at the center of the carotenes [14,17]. Retinal can be further reduced by retinal reductase to retinol. Lycopene is a non-pro-vitamin A carotenoid and has been shown to possess anti-cancer activity as demonstrated in laboratory tests. Although there are several hundreds of carotenoids in natural source, approximately only 50 of them are present in common vegetables and fruits in US diets [16,18]. Finally, perhaps just over a dozen of these carotenoids or their metabolites can be detected in human blood and tissues which may be attributed to human health [16,18].

Anti-PC activities of carotenoids and vitamin A are thought to rely on their antioxidant activities. Excessive reactive oxygen (ROS) and nitrogen species (RNS) can be produced during aerobic metabolism and pathological processes and then cause damages in cellular lipids, DNA or proteins [17,19,20]. Carotenoids are usually involved in the scavenging of two types of the ROS, singlet molecular oxygen (^1^O_2_), and peroxyl radicals. Carotenoids can directly transfer excitation energy from ^1^O_2_ and generate a ground state oxygen and an excited triplet state carotenoid, which further transforms the energy into heat and the ground state intact carotenoid [14,21,22]. The potency of this kind of quench effects is closely related to the number of conjugated double bonds of a given carotenoid, thus lycopene is the top quencher among carotenoids. Carotenoids like lycopene can also trap other ROS and RNS, like ·OH, ·NO_2_ or peroxynitrite, however, leading to oxidative breakdown of the carotenoid molecules [22,23,24,25]. This action seems to suggest that the carotenoids may act in the front line of defense. It has been demonstrated that lycopene can reduce oxidative DNA damage in cell cultures and in rats *in vivo* [26,27]. Moreover, human studies showed that tomato consumption could protect human leukocytes against oxidative DNA damage [28]. Studies [29] also showed that a lycopene-rich tomato sauce consumption could reduce oxidative DNA damage in human prostate and PC.

Another related anticancer activity of carotenoids and retinoids is that they can increase the expression and function of a number of antioxidant enzymes and detoxifiying enzymes [30,31]. It has been shown that carotenoids tested, especially lycopene may up-regulate phase II detoxification enzymes through activation of Nrf2 transcription factor and its binding to the antioxidant response element (ARE) of phase II detoxification enzyme genes. The studies showed that lycopene induced an increase of the phase II enzymes NAD(P)H:quinone oxidoreductase and gamma-glutamylcysteine synthetase at mRNA and protein levels in human cancer cell lines examined.

In addition, other anti-cancer activities of β-carotene and other carotenoids have been demonstrated in several non-prostate cancer cell lines. Several mechanisms may account for proapoptotic effects of β-carotene such as induction of caspase cascade [32,33], disruption of mitochondrial functions [32], alterations of apoptosis-related proteins, including Bcl-2 and Bcl-xL [33], Bad [34], Bid [34] and Bax [35,36] and FAS [37,38]. Moreover, β-carotene may also increase the levels of transcription factors related to apoptosis induction [39] including c-myc [40] and Nuclear Factor Kappa-B activation [40]. However, anti-proliferative effects of β-carotene on PC cell lines tested was questioned in the study by Dulinska J. *et al*. [41]. For clarification, a recent study [42] showed that β-carotene can cause apoptosis only in caveolin positive PC cells but not caveolin negative cells, indicating caveolin-1 is the target of β-carotene for cell death. Again, whether all β-carotene activities mentioned above are acted through its antioxidant activities is not all clear.

Retinoids are important factors for early normal development and cell differentiation. Retinoic acids and retinol in our body system must be obtained from pro-vitamin A carotenoids or preformed retinoids [14,16,43]. Anti-cancer effects of retinoic acids are mainly mediated *via* two classes of nuclear receptor family, *i.e.*, retinoic acid receptors (RARs) α, β, γ and retinoid X receptors (RXRs) α, β, γ [43,44]. In fact, Retinoids play an important role in regulation of normal prostate growth and differentiation [45,46]. Mouse knockouts of RAR α and γ showed metaplasia and keratinization in the prostate and prostatic lumen defect in secretion. In laboratory studies, retinoids can induce cell cycle arrest, inhibit PC proliferation as well as reduce prostate carcinogenesis [46]. Retinoids may down-regulate anti-apoptotic Bcl2 and upregulate RARβ and tissue transgluaminase in prostate cancer cells [47]. Retinoic acid was also shown to inhibit the function of the androgen receptor [48]. In fact, RAR β2 [49] and retinoic acid synthesis gene ALDH1A2 [50] have been viewed as tumor suppressor genes. The promoter regions of these two genes are found to be highly methylated in primary PC tissues, resulting in lowered expression in tumor tissues compared to normal prostatic tissues. Re-activation of these genes can be demonstrable by using demethylating agents (e.g., 5’aza-deoxycytidine) [60,61,62]. One can speculate that carotenoids might enhance efficacy of the epigenetic therapy using demethylating agents for treating PC if RARβ2 and ALDH1A2 are silenced by aberrant DNA methylation.

Although most of laboratory results, including animal studies, indicated that carotenoids, vitamin A and retinoic acids are promising dietary compounds for reducing PC risk, related human studies from many observational epidemiological or clinical trial reports show inconsistent or conflicting results. For example, some early epidemiologic studies suggested that increasing consumption of tomatoes or tomato products was significantly associated with decreased PC risk [52,53,54]. Lycopene in tomato was suggested to be the main factor for reducing PC risk. Other observational or trial studies found that the degree of the inverse association of lycopene or plasma levels of α-carotene, β-carotene and lycopene with PC risk was from moderately significant or modest but not significant to none [55,56,57,58]. A case control study [59] with 118 nonmetastatic PC non-Hispanic Caucasian men and 52 healthy men from southeast Texas was conducted to assess any association between cancer risk and plasma levels of several specific types of carotenoids. The results showed that increased levels of β-cryptoxanthin, α -carotene, trans-β-carotene, lutein and zeaxanthin in these men may be associated with reduced PC risk. A population-based case-control study in Arkansas with 193 PC cases and 197 matched controls [60] was conducted to measure plasma levels of lycopene, lutein/zeaxanthin, and β-cryptoxanthin. The data indicated that the levels of these carotenoids were inversely associated with PC risk. In the European Prospective Investigation into Cancer and Nutrition study [61], plasma from 966 PC cases and 1064 control subjects were obtained to determine the levels of seven carotenoids, retinol, α-tocopherol (a vitamin E), and γ-tocopherol (a vitamin E). The results showed that there were no associations between plasma concentrations of carotenoids, retinol, or tocopherols and overall PC risk. However, the authors did indicate that there were some inverse associations of lycopene and the sum of carotenoids with the risk of advanced PC disease. A more recent study by a nested case-control design with 467 PC cases and 936 cancer free controls [62] was conducted for measuring serum antioxidant levels such as selenium, tocopherols, carotenoids and retinol. It found no association of these serum antioxidants with overall PC risk. Another recent nested case-control study [63] with 692 PC cases and 844 matched controls from the Prostate, Lung, Colorectal, and Ovarian Cancer Screening Trial was conducted to investigate if serum concentrations of retinol were associated with PC risk. Again, it showed no association of serum levels of retinol with overall PC risk, yet higher serum retinol levels were associated with a lower risk of aggressive PC.

There were clinical trials to test carotenoid effects on reducing PC risk. The β-Carotene and Retinol Efficacy Trial (CARET) is a randomized, double-blind, placebo-controlled, lung cancer chemoprevention trial of β-carotene 30 mg and retinol 25,000 IU/day [57]. This prospective nested case-control study was performed to determine the association between serum carotenoids, retinoids, and tocopherols on both lung and prostate cancer incidence. Unfortunately, the authors did not find any significant association of the serum carotenoids and PC risk. In addition, with a longer follow-up (an average of 11 years) of 890 PC cases, the newer report [64] from the CARET showed consistent results that no association of dietary supplement use and PC risk. Part of the Alpha-Tocopherol, Beta-Carotene Cancer Prevention (ATBC) Study cohort of Finnish male smokers [65] was used to determine the relationship between a family history of PC and PC risk in relation to uptake of α-tcopherol and β-carotene micronutrients. The conclusion was that the authors did not find that supplementation with vitamin E or β-carotene can modify the family history-prostate cancer association. In a separate ATBC study report, the authors [66] found that serum β-carotene, serum retinol, and supplemental β-carotene did not seem to have effects on PC patient survival. Also, note, intervention of daily β-carotene supplements (20 mg and 30 mg, respectively) may increase lung cancer risk among smokers as reported in the ATBC and CARET studies [57,65].

Overview of epidemiological and intervention trial studies for vitamin A and related compounds: There are 15 reports [52,53,54,55,56,57,58,59,60,61,62,63,64,65,66] mentioned above. Three presented that tomato or tomato products may have protection effects for PC. Among the remaining 12 studies [55,56,57,58,59,60,61,62,63,64,65,66], only four [55,56,59,60] showed plasma carotenoids or carotene supplements had effects to reduce PC risk.

## The B Vitamins

One carbon metabolic pathway has important roles in DNA synthesis, repair and methylation, in which a carbon unit from serine or glycine is transferred to tetrahydrofolate (THF) to form 5-methylene-THF by a key enzyme methylene-THF reductase (MTHFR). In addition to folate (also called vitamin B_9_), other B vitamins such as riboflavin (vitamin B_2_), vitamins B_6_ and B_12_ also serve as coenzymes in the folate mediated one carbon pathway [67,68,69]. Methylene-THF can be further used as a precursor for forming building blocks for DNA or RNA synthesis or reduced to methyl-THF which in turn donates a methyl group to methionine to form *S*-adensoylmethionine (SAM) by a B_12_-containing methyltransferase. SAM, in fact, is a universal donor of methyl groups for DNA methylation and many other cellular reactions. Thus it is thought that these B vitamins are involved in regulating DNA metabolism pathways and may be related to cancer development or progression.

Although it has been proposed [70,71] that there may be certain associations between intakes or circulating levels of these B vitamins and the risk of PC, epidemiologic studies [70,71,72,73] showed conflicting results. For example, a prospective cohort study reported an association of decreased folate levels and increased risks of PC mortality [70]. A case-control study [71] with 1,294 PC patients from various areas of Italy showed that high intake of dietary folate might reduce PCa risk. On the other hand, Hultdin *et al*. [72] examined plasma levels of folate and vitamin B_12_ in a prospective study of 254 case subjects and 514 matched control subjects and found that increased folate and vitamin B_12_ levels in plasma were statistically significantly associated with increased PCa risk. Another large prospective study [74] found that increased plasma concentrations of vitamin B_2_ were associated with an increased PC risk. The American Cancer Society Cancer Prevention Study 11 Nutrition Cohort study [73] conducted a study for the association of folate intake with PC among 65,836 US. men in a nine years follow-up, and the survey did not find an association of dietary or total folate intake with the overall PC risk. A placebo-controlled randomized trial of aspirin and folic acid supplementation for cancer chemoprevention in the Aspirin/Folate Polyp Prevention Study [75] was conducted with up to about 10 years follow up. Secondary findings of this trial regarding PC incidence were reported that, although aspirin showed no correlation with PC incidence, folate supplement intake had a positive association with increased PC risk. However, interestingly, baseline dietary folate was inversely associated with PC risk. The authors concluded that the effect of supplemental folate on PC risk may be different from that of dietary folate, indicating a complex role of folate metabolism in PC. Furthermore, a study with two randomized, double-blind, placebo-controlled B vitamins intervention trials, *i.e.*, the Norwegian Vitamin Trial and the Western Norway B Vitamin Intervention Trial with a total of 6,837 patients having ischemic heart disease was conducted between 1998 and 2005, plus two additional years follow up. The oral treatment groups of the study included folic acid (0.8 mg/d) plus vitamin B_12_ (0.4 mg/d) and vitamin B_6_ (40 mg/d), folic acid (0.8 mg/d) plus vitamin B_12_ (0.4 mg/d), vitamin B_6_ alone (40 mg/d), and placebo. The authors indicated that the given dose of folic acid were twice the recommended daily allowance but below the tolerable upper intake level of 1 mg/d [76]. The conclusion of the study was that the treatment with folic acid and vitamin B_12_ may increase cancer incidence including PCa and all-cause mortality. The authors further commented that high doses of folic acid might enhance the growth of established neoplastic lesions, perhaps in part, because excessive unmetabolised plasma folic acid may have a potential to reduce natural killer cell activity [77] with subsequent weakened cancer immunity.

In addition to vitamin B intakes, genes coded for the enzymes in the one carbon metabolism pathway could be another factor to influence efficiency of use of the nutrients. For example, it was suggested [78,79] that genetic polymorphism of the MTHFR gene may have a potential implication in the relationship of dietary folate intake with PC risk. There are studies [80,81,82] that showed certain single nucleotide polymorphisms (SNPs) in the MTHFR gene may protect PC risk. However, other studies [83,84] examing SNPs in the MTHFR gene and other genes in the one carbon metabolism found no such an association.

Overview of epidemiological and intervention trial studies for B vitamins in one carbon metabolism: Among 8 reports [70,71,72,73,74,75,76] discussed above, two of them showed an inversed association of folate intake with PCa risk. Four of them indicated that one or two B vitamins could increase PC risk. Only one report showed no effects of B vitamins on PC risk. In addition, there are seven reports [77,78,79,80,81,82,83] regarding genotypes or SNP analysis of the MTHFR gene or other genes in one carbon metabolism related pathways, five of them showed SNPs had effects on PC risk.

## Vitamin C

Vitamin C or ascorbic acid is an essential nutrient for humans in collagen, carnitine and neurotransmitter biosyntheisis. Because of lacking a key enzyme gulonolactone oxidase for synthesizing ascorbic acid, humans have to rely on its supply from foods. Although its anti-cancer activities have been realized, whether the activities are due to vitamin C’s anti-oxidant or pro-oxidant ability remains an open question [85]. Besides its anti-oxidant/ pro-oxidant activities, there was little information about other potential anti-PC mechanisms for vitamin C except that the expression and function of the androgen receptor was shown to be inhibited by vitamin C in a PC cell line [86]. Currently, even with many observational epidemiologic and clinical trial studies, whether vitamin C has a potency to reduce PC risk remains uncertain. For example, the SU.VI.MAX trial [87] was conducted with 5,141 men randomized for receiving a placebo or a supplementation with nutritional doses of vitamin C, vitamin E, β-carotene, selenium and zinc daily for 8 years. The authors only found a moderate nonsignificant inverse association of PC risk with the supplementation. However, the study indicated that there was a marked statistically significant reduction in PC incidents for those men with normal PSA levels and receiving the supplements. Another trial [88] with supplemental and dietary vitamin E, beta-carotene, and vitamin C intakes for up to eight years follow up, did not find any effect of these antioxidant supplementation on PC prevention. A small prospective study [89] showed prediagnostic plasma vitamin C levels were not associated with reduction of PC risk. A few recent observational studies [90,91,92] investigating the role of dietary intake of vitamin C and other dietary antioxidants showed some inverse association of vitamin C intake with PC risk. Nonetheless, a recent, large clinical trial study [93] designed for further proof of PC chemoprevention efficacy of vitamin C did not produce any supportive result.

Recently a controversial issue regarding whether vitamin C can be effectively used to treat cancers including PC rises. It has been shown that injection of mega doses of vitamin C can reach its pharmacologic levels in circulation that can not be achieved by oral administration [94,95,96,97]. Studies [98,99] showed the pharmacologic levels of vitamin C may selectively toxic to cancer cells. In vitro cell culture study showed that ascorbate at pharmacologic concentrations acted as a prooxidant, producing hydrogen-peroxide-dependent cytotoxicity in a variety of cancer cells without affecting normal cells. In glioblastoma xenografts hydrogen peroxide was formed within interstitial fluids of tumors but not in blood with intravenous (IV) administration of pharmacologic ascorbate. In addition daily pharmacologic ascorbate treatment showed significantly decreased growth of ovarian, pancreatic, and glioblastoma tumors in xenograft mice. Other studies [100,101] also showed that anti-proliferative effects of pharmacologic ascorbate may act through inhibition of angiogenesis or inhibition of genes necessary to cell cycle progression in two tumor models. Two phase 1 clinical trials [102,103] of vitamin C on cancer seemed to indicate well tolerance and safety of high dose iv. administration of vitamin C to cancer patients. Renewed interest in vitamin C for cancer treatments is increase, but its skeptics also exists [104]. Another issue is that vitamin C may induce antagonistic effects on routinely used antineoplatic drugs [105].

Overview of epidemiological and intervention trial studies for vitamin C: In nine reports [87,88,89,90,91,92,93] mentioned, only three [90,91,92,93] showed vitamin C had protection effects on PC risk.

## Vitamin D

Vitamin D_3_ (cholecalciferol), a prohormone of vitamin D is synthesized in skin cells from 7-dehydrocholestrol by exposed to ultraviolet B radiation of sunlight. Vitamin D_3_ or vitamin D_2_ (ergocalciferol), another vitamin D proform derived from fungus products, is metabolized to calcidiol 25(OH)D_3_ in the liver and then converted to calcitriol 1,25(OH)_2_D_3_, a biologically active vitamin D, by 1α-hydroxylase in the kidney and other tissues, including prostate [106,107,108,109]. Usually circulating [25(OH)D_3_] level is used to determine vitamin D nutritional status. Antcancer activities of vitamin D have been suggested to act mainly through its nuclear receptor or vitamin D receptor. It has been shown [106,107,110,111] that vitamin D may cause PC cellular dysfunctions or altered gene expression including inhibition of cell proliferation, cell invasiveness, angiogenesis and c-Myc and telomerase expression, or induction of cell differentiation and apoptosis. Other PC cell growth inhibition mechanism by vitamin D may be from its ability to alter the expression of some key genes in the metabolism of prostaglandins (PGs) such as inhibition of the expression of the PG synthesizing cyclooxygenase-2 and the PG receptors EP2 and FP, and increased expression of PG inactivating 15-prostaglandin dehydrogenase [112]. Alteration of these gene expressions would decrease the cell proliferative stimulus of PGs in PC cells.

Vitamin D deficiency or insufficiency has become a public health concern in large proportions of the populations in the United States and Northern European countries especially among ethnic groups with dark skin, and others such as those with physical inactive and little sun exposure. Prolonged sun light exposure without adequate skin protection can cause skin cancer, but proper sun exposure may be beneficial to protect from some types of cancer, including PC. Indeed, many epidemiologic studies showed [109,113,114,115,116] a high degree of consistent results that sun light exposure is inversely associated with PC risk. This protection effect is thought due to the effect of vitamin D production. Yet not every study was fully support this idea. For instance, a population-based nested case-control study and meta-analysis [117] only provided a limited support for the effect of sunlight on reducing PC. Moreover, when using serum vitamin D levels to determine the association, the protection effects seem to be less consistent than that using sunlight exposure for studies. Again, some studies [107,109,113,114,115,116,118] support the notion that high levels of serum vitamin D have protection effects, others [120,121] showed different conclusions. As reported in two recent studies [122,123], vitamin D intake was not associated with reduction of PC risk. A large nested case-control study with an European population [120] indicated no beneficial effects of blood vitamin D levels for reducing PC risk. Another recent large prospective study [122] also did not show vitamin D has effects on reduction of PC risk. On the other hand, the same report stated that higher blood 25(OH)D levels may be related to increased risk of aggressive PC. Since functions of the vitamin D receptor (VDR) and related vitamin D metabolic enzymes are associated with vitamin D’s action, a report [124] showed that several single nucleotide polymorphisms (SNP) in the 3’-untranslated region of the VDR gene, not other genes, are associated with PC risk in men having low blood vitamin D levels. Other studies also indicated that different SNPs in the VDR gene may be associated with lowering PC risk [125,126,127,128]. Still, there are studies [108,129,130,131] producing otherwise or unclear results. Overall, whether genetic variants of those genes involved in vitamin D pathways have real effects on PC risk remains conflicting and requires more studies.

Although it has been suggested that vitamin D (*i.e.*, calcitriol) may be used as a therapeutic agent for PC, its side effect of hypercalcemia and hypercalciuria at the pharmacologic doses has restricted its clinical usage [132]. Several approaches have been used to circumvent the potential side effects of calcitriol. One such approach is to develop new analogues with reduced calcemic side effects [133,134]. For example, 19-nor-1α,25-dihydroxyvitamin D_2_ has reduced calcemic effects with a similar anticancer activity to calcitriol [134]. A newer analog, 19-nor-2α-(3-hydroxypropyl)-1α,25-dihydroxyvitamin D_3_ (MART-10) was shown to be more effective in anti proliferation activity for an immortalized normal prostate cell line (1,000-fold) and anti-invasion activity of PC-3 PC cell line (10-fold), when compared to calcitriol [134]. Clinical trials will require to demonstrate the safety and therapeutic efficacy of these analogs for PC treatment.

Investigators also examined if low doses of calcitriol in combination with other anticancer agents can be used for effective PC treatment. An example [135] is that in a recent preclinical study the authors showed that combination of low dose calcitriol and RRR-α-vitamin E succinate has a superior antitumor activity in cell culutres and a xenograft model to single agents used. It has also been suggested [136,137] that combining with other antineoplstic durgs such as carboplatin, taxanes, or dexamethasone may decrease doses of calcitriol, therefore reduce the toxicity of calcitriol and enhnace cancer killing effcts of the drugs used. Moreover, an example of another type of PC therapy strategies [138,139] is to use weekly, instead of daily, oral administration of high dose calcitriol (*i.e.*, DN-101 formulation) to allow to maintain effective antitumor levels of serum calcitrol concentrations with reduced side effects [140]. The authors used this formulation with docetaxel for treating PC patients in Phase II and Phase III trials [141,142]. The calcitriol treatment formulation seemed not to increase toxicity to patients with some promising results pending further confirmation.

Overview of epidemiological and intervention trial studies for vitamin D: Four [109,113,114,115] of six studies [109,113,114,115,116,117] with sunlight exposure effects showed protection against PC. Eight [107,109,113,114,115,116,118] of eleven reports [107,109,113,114,115,116,118,120,121,122,123] regarding serum vitamin D levels or intervention trials demonstrated that vitamin D had protection effects for PC. For the genetic effect of the vitamin D receptor, five [124,125,126,127,128] out of nine reports [108,124,125,126,127,128,129,130,131] indicated that SNPs of the receptor gene had effects on PC risk.

## Vitamin E

Vitamin E, as an antioxidant, consists of at least eight isoforms of naturally occurring compounds in diets including α-, β-, γ-, and δ- tocopherols and α-, β-, γ-, and δ- tocotrienols [143]. Since it is the most abundant natural vitamin E, α–tocopherol has been used in evaluating its efficacy in reducing PC risk. Two most recent large vitamin E studies, The Physicians’ Health Study II Randomized Controlled Trial [93], and Selenium and Vitamin E Cancer Prevention Trial [SELECT] [144] were reported with 14,641 male physicians in the United States for vitamin E and C and with 35,533 men of a general population for vitamin and selenium on Prostate cancer risk, respectively. These two large randomized, placebo-controlled trials were motivated by strong positive results from previous studies such as the Finnish ATBC (α-Tocopherol, β-Carotene) Cancer Prevention Trial [56] and designed to once for all to prove the effects of the dietary antioxidants on PC. However, the outcomes of these two most recent reports showed vitamin E or vitamin E plus selenium at the doses and formulations used, did not reduce PC risk in a selected population or a relatively healthy male population. Interestingly, the SELECT Trial study showed that selenium had statistically nonsignificant effect on increase of type 2 diabetes mellitus.

Obviously the above two large scale studies seem to confirm some previous studies [88,145,146] whose findings demonstrated no association of intake of vitamin E or serum α-tocophenrol levels with PC risk. However, one of the latter studies did indicated that vitamin E supplementation in male smokers and β-carotene supplementation in men with low dietary β-carotene intakes seemed to be associated with reduced risk of this disease. On the other hand, there are reports [59,147,148,149] showed benefit against PC. Even though, whether further studies will be needed to settle the disputes from these studies still remains open. However, some studies [150] suggested that γ-tocopherol may be more relevant than α–tocopherol to the protection by vitamin E against PC.

Like α–tocopherol, γ-tocopherol is also a major vitamin E detected in human circulation. It was shown that γ-tocopherol may have a higher efficacy than α–tocopherol to protect against lipid oxidation induced by peroxynitrite, a strong mutagenic oxidant [151,152]. Also, it has been shown that γ-tocopherol may have stronger anti-inflammatory effects as well as stronger anti-proliferation and apoptotic induction effects in many cancer cell lines than α–tocopherol [153]. Another potential anti-cancer activity of γ–tocopherol is its ability to up-regulate peroxisome proliferator-activated receptor-γ, a nuclear receptor capable of inhibiting cell proliferation or inducing cell death. Using a transgenic rat prostate cancer model (TRAP), the authors [154] showed γ-tocopherol in the diet can induce activation of caspases 3 and 8 in the ventral prostatic lobes and repress progression from prostatic intraepthilelial neoplasia to adenocarcinoma. Another study [155] with a transgenic mouse prostate cancer model (TRAMP) γ-tocopherol-enriched mixed tocopherol diet significantly up-regulated the expression of Nrf2 and its related detoxifying and antioxidant enzymes thereby suppressing PIN and tumor development. A recent large prospective study [156] by examining 295,344 American men for the association of prostate risk with supplemental vitamin E comsumption and dietary intakes of α-, β-, γ-, and δ-tocopherols. The study showed that supplemental vitamin did not reduce PC risk, however, increases in γ-tocopherol uptake was significantly inversely associated with the risk of advanced PC.

Overview of epidemiological and intervention trial studies for vitamin E/α-tocopherol: Five [88,93,144,145,146] out of elevan reports listed showed no effects, the rest [59,147,148,149,156] indicated protection effects.

## Conluding Remarks

As discussed above, multiple studies showed the aforementioned vitamins have positive, negative or null effect on reducing PC risk. Even intervention studies can not absolutely determine if these vitamins have anti-PC efficacies. Note, two excellent studies [76,157] actually showed that use of anti-oxidants (e.g., carotene, vitamin A, vitamin E) and B vitamins (e.g., folic acid and B_12_) in intervention trials may potentially increase both cancer and/or an all-cause mortality rate. Too many factors can affect the outcomes or interpretations of the studies, including extricate methodolgies for studies (e.g., statistical methods, detection technologies, *etc*.,) and intrinsic knowledges and understandings of vitamins in each individual human body (e.g., absorption rates, transportation, metabolic pathways, genetic and epigenetic factors, *etc*.). In addition, not only these vitamins but also many other potential anti-cancer micronutirents or dietary factors coexist or are consumed in our bodies, it becomes very complicated and problematic when studying one without consideing others (or just holding others as if they are constant as at baselines). Conceivably, these researches will continue as needed, using more new advanced technologies and knowledges.
